# Evaluation of three ELISA assays for detection of *Mycobacterium avium* subsp. *paratuberculosis* antibodies in serum of sika deer (*Cervus nippon*)

**DOI:** 10.1038/s41598-026-51629-1

**Published:** 2026-05-19

**Authors:** Nasser Alotaibi, Emily A. Courcier, Máire C. McElroy, Simon Liggett, Hanne Jahns

**Affiliations:** 1 Department of Pathology, College of Veterinary Medicine, King Faisal University, Al-Ahsa, 31982 Saudi Arabia; 2 Pathobiology Section, School of Veterinary Medicine, Veterinary Science Centre , University College Dublin, Belfield, Dublin 4, D04W6F6 Ireland; 3 Centre for Veterinary Epidemiology and Risk Analysis, University College Dublin, Dublin, Ireland; 4 Central Veterinary Research Laboratory, Department of Agriculture, Food and the Marine, Celbridge, Kildare, Ireland; 5 Disease Research Ltd, Mosgiel, New Zealand

**Keywords:** ELISA, Faecal culture, Farmed sika deer, Johne’s disease, Sensitivity, Specificity, Biological techniques, Diseases, Immunology, Microbiology, Zoology

## Abstract

ELISA-based assay testing offers a practical, high-throughput approach for early detection of *Mycobacterium avium* subsp. *paratuberculosis* (MAP), the causative agent of Johne’s disease (JD), particularly for identifying subclinically infected animals within a herd. As no ELISA has been developed specifically for sika deer, the aim of this study was to compare the diagnostic performance of two commercial ELISA assays (ID.VET and IDEXX) designed for cattle, sheep and goats, and the Paralisa™ assay developed for red deer. Serum and faecal samples (*n* = 105) were collected from 36 clinically affected sika deer at postmortem, 40 apparently healthy sika deer from the same infected herd at slaughter, and 29 sika deer from a JD-free herd at postmortem. Individual liquid faecal culture confirmed infection and MAP shedding status, with 73 animals (69.5%) testing culture positive. Sensitivities were 48% (Paralisa™), 43% (ID.VET) and 4% (IDEXX), while all assays showed 100% specificity. Paralisa™ and ID.VET assays demonstrated almost perfect agreement (κ = 0.86), whereas the IDEXX assay showed poor agreement with both assays. ID.VET assay also showed excellent inter-laboratory repeatability (ICC = 0.93). Overall, Paralisa™ and ID.VET assays provided superior sensitivity for MAP antibody detection in sika deer and may aid JD monitoring in infected herds.

**Supplementary Information:**

The online version contains supplementary material available at 10.1038/s41598-026-51629-1.

## Introduction

Johne’s disease (JD), also known as Paratuberculosis (PTB) caused by *Mycobacterium avium* subsp. *paratuberculosis* (MAP), is a chronic granulomatous enteritis of farmed and wild deer reported in various regions worldwide that results in progressive weight loss, diarrhoea, and eventually death^1–3^. In farmed deer, JD is of increasing concern due to its impact on animal health, productivity, and farm profitability. Economic losses arise not only from mortality but also from reduced growth rates, decreased carcass value, and costs associated with diagnostic testing and herd management^4^. Transmission occurs predominantly via the faecal-oral route, where young deer are exposed through contaminated feed, water, or pasture. Subclinical infection in deer plays a vital role in the epidemiology of MAP, as these animals shed bacteria intermittently while remaining clinically normal, thereby acting as hidden reservoirs of infection within the herd^2,5,6^. Therefore, the early detection and management of such subclinical carriers are especially difficult, making control of JD highly challenging in deer production systems.

A variety of diagnostic methods have been developed to identify MAP-infected animals, each with advantages and limitations. Bacterial culture of faeces and tissues remains the reference standard method for diagnosing JD in farmed deer species, as it directly detects viable MAP organisms and provides information on bacterial load and shedding patterns^2,6,7^. However, culture is time-consuming, prone to contamination, and has limited sensitivity, particularly in subclinically infected deer where bacterial excretion is often low or variable making detection challenging^2,6,8^. A Bayesian latent class analysis in farmed red deer (*Cervus elaphus*) estimated the overall sensitivity of individual liquid faecal culture at 77% (95% CI: 61–92%) and the specificity at 99% (95% CI: 98–99.7.7%), confirming its high accuracy but limited ability to detect all subclinically infected deer^9^. In contrast, in white-tailed deer (*Odocoileus virginianus*), faecal culture failed to recover MAP on slant due to heavy bacterial and fungal overgrowth, rendering the cultures diagnostically unusable^8^.

As an alternative, antibody-based enzyme-linked immunosorbent assays (ELISAs) provide rapid, cost-effective, and high-throughput screening suitable for herd-level surveillance^10–13^. Despite their practicality, ELISA results are highly dependent on host species, the immunological stage of infection, antigens used and assay characteristics, and thus their performance can vary considerably^9,14–16^. In subclinical infection, immune responses are predominantly Th1-mediated, favouring cellular immunity but resulting in limited antibody production and consequently low ELISA sensitivity^17–21^. As animals progress to clinical disease, a shift toward Th2-dominated humoral immunity occurs, leading to increased antibody titres and improved serological detectability^17–20^.

Commercial ELISA assays such as ID.VET and IDEXX were primarily developed for cattle, sheep and goats, and although widely used in these species, their diagnostic accuracy in deer remains less certain^21–25^. This limitation has prompted the development and evaluation of deer-adapted ELISAs, which use species-specific conjugates to improve diagnostic performance. In fallow deer (*Dama dama*), an in-house ELISA using deer-specific conjugates achieved sensitivities of 72% with 100% specificity for animals with lesions consistent with JD at necropsy, whereas a commercial assay (ID.VET) designed for cattle, sheep, and goats showed comparable sensitivity (~ 72%) but lower specificity (87.5%)^26^. In elk (*Cervus canadensis*), modification of the IDEXX assay by replacing the anti-bovine conjugate which has poor affinity for elk, bison, and caribou IgG with protein G or an anti-deer conjugate improved diagnostic performance, yielding sensitivities of 73% (protein G) and 68% (anti-deer), with specificities of 90% and 100%, respectively, after recalibration of cut-off values^10^. The value of species-specific assays is further illustrated in farmed red deer, where the IgG1 ELISA commonly known as Paralisa™ reported sensitivities up to 84–91% and specificities around 99.5% in clinically affected red deer^27^, and 19% with specificity 94% in subclinically infected deer^9^.

Further, appropriate ELISA assays are needed for EU required JD surveillance in zoo and wild ruminants, which have been listed as potential reservoirs, including sika deer, in the Animal Health Law (Regulation EU 2016/429) on transmissible animal diseases^28^. In addition, recent MAP surveys conducted in zoo and wildlife populations including cervids and other herbivores have identified subclinically affected animals, highlighting their potential role in spreading the disease^29,30^.

Studies of JD caused by MAP in sika deer remain exceptionally limited compared with domestic ruminants. Only a few isolated case reports have been documented worldwide, including the first description in a park-maintained sika deer^31,32^, clinical disease in captive sika deer in Ohio, USA^33^, and a detailed case in a three-year-old stag^34^, largely diagnosed by histopathology and acid-fast staining. Beyond these reports, a serological survey in farmed sika deer in China identified evidence of MAP exposure at the herd level, providing limited epidemiological data for this species^35^. Collectively, these findings highlight both the susceptibility of sika deer to MAP infection and the scarcity of published data.

The diagnostic performance of commercial non-deer-specific ELISAs relative to the Paralisa™ assay designed for red deer has not previously been assessed in sika deer. Therefore, performance of three ELISA assays (ID.VET, IDEXX, and Paralisa™) for the detection of MAP-specific antibodies was evaluated and compared in terms of sensitivity, specificity and agreement in subclinically and clinically MAP infected farmed sika deer, using faecal culture as the gold standard diagnostic method. By providing a comparative evaluation, this study addresses a critical knowledge gap in the diagnosis of JD in this deer species and supports the selection of appropriate serological tools for disease monitoring and control in sika deer.

## Results

### Distribution of MAP culture results by disease status

**Fig. 1:**
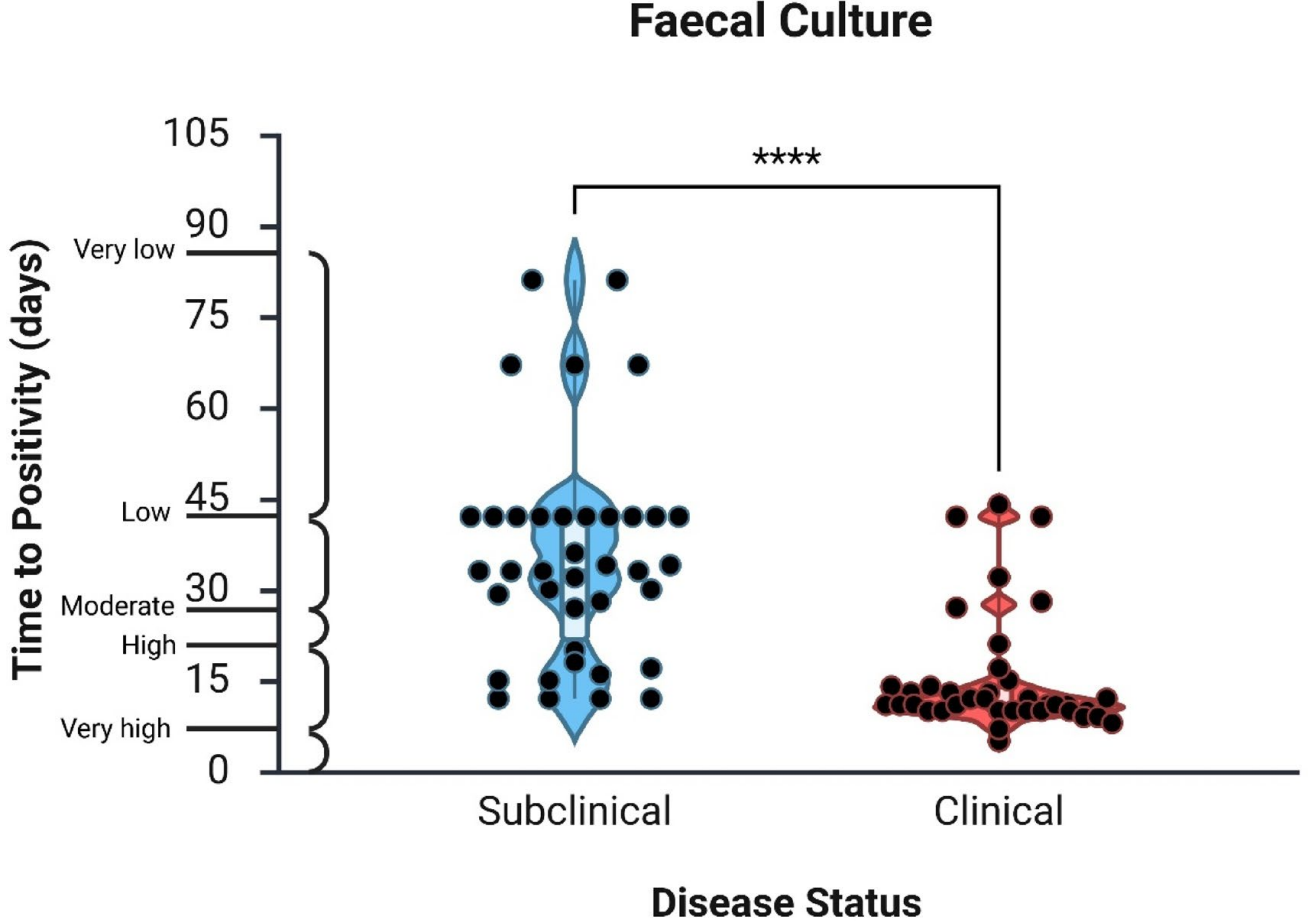
A violin plot of the faecal culture results (time to positivity). It revealed a significantly greater shedding rate in clinically infected sika deer than in subclinically infected sika deer. Each spot represents one animal tested. Statistical significance was determined via the two-tailed Mann-Whitney U Test, ****p < 0.0001. Created with BioRender.com

Faecal culture was used as the gold standard method for defining MAP infection status. As expected, all animals from JD-free herds (*n* = 29) were culture negative, confirming their MAP-free status (Supplementary Table 1). The subclinical group (*n* = 40) demonstrated variable culture positivity. While three animals (7.5%) were culture negative, the remaining 37 (92.5%) showed detectable bacterial growth, most frequently at low levels (*n* = 21, 52.5%). Smaller proportions displayed very low (*n* = 5, 12.5%), moderate (*n* = 2, 5%), or high (*n* = 9, 22.5%) bacterial loads (Fig. 1, Supplementary Table 2). Clinically affected deer (*n* = 36) had the highest culture positivity, with all 36 (100%) testing positive. The shedding rates of clinically affected deer were significantly higher when compared to subclinical deer, as confirmed by a Mann-Whitney U test (*p* < 0.0001). The majority yielded high (*n* = 28, 77.8%) or very high (*n* = 1, 2.8%) bacterial growth, while only a few exhibited moderate (*n* = 3, 8.3%) or low (*n* = 4, 11.1%) loads (Fig. 1, Supplementary Table 3).

### Serological detection by ELISA assays

**Fig. 2:**
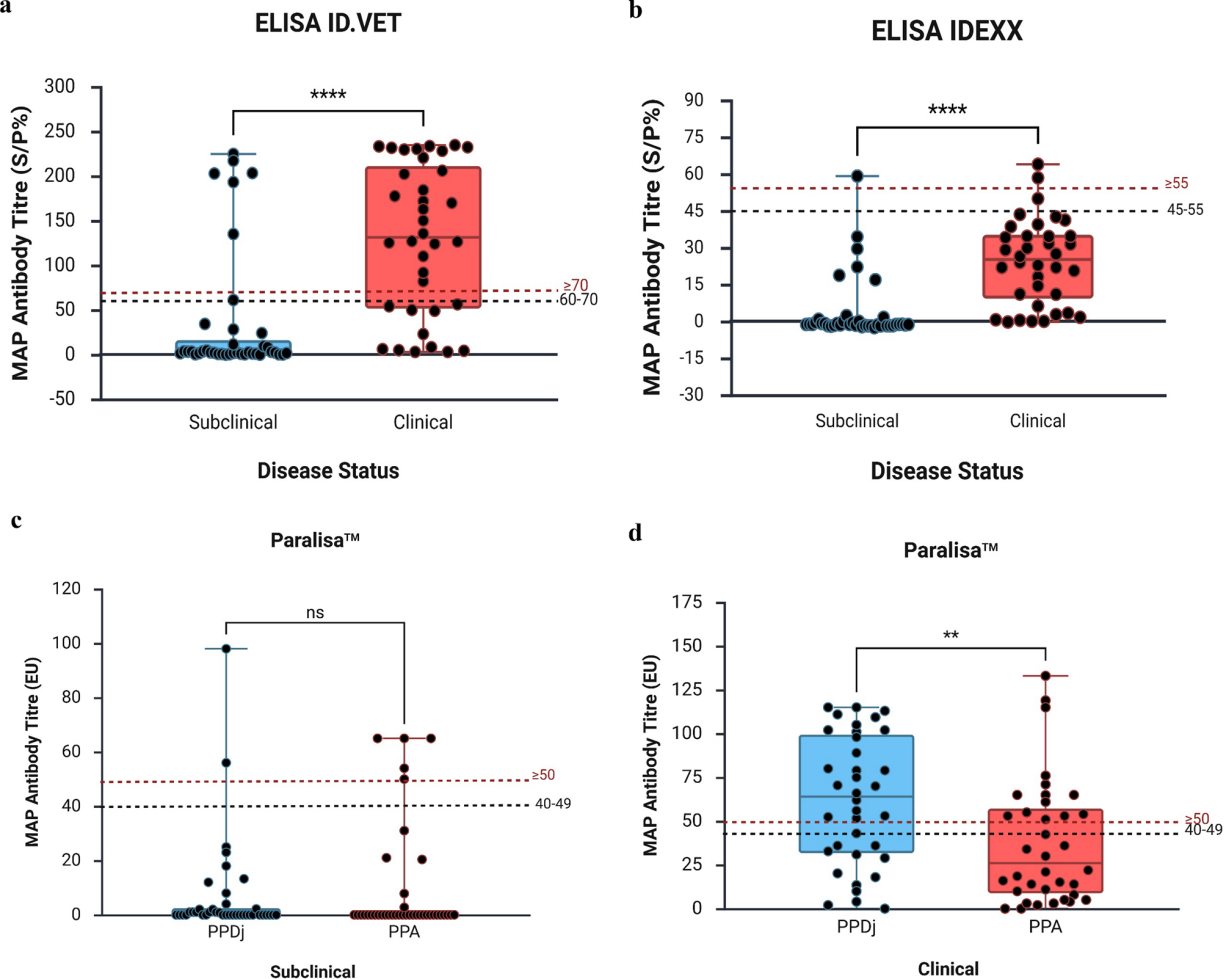
Box and whisker plot showing the detection of antibody titres to MAP by three different ELISAs. The dashed line (red) indicates the positive cut-off value of each test. The dashed line (black) indicates the suspect (doubtful) cut-off value of each test. Each spot represents one animal tested. **a** ID.VET ELISA antibody titres in subclinical and clinical MAP-infected sika deer (≥ 70% cut-off). **b** IDEXX ELISA antibody titres in subclinical and clinical MAP-infected sika deer (≥ 55% cut-off). **c** Paralisa™ (PPDj and PPA) ELISA antibody titres in the subclinical group (≥ 50EU cut-off). **d** Paralisa™ (PPDj and PPA) ELISA antibody titres in the clinical group (≥ 50EU cut-off). Statistical significance was determined via the two-tailed Mann-Whitney U Test, *p < 0.05, **p < 0.01, ***p < 0.001, ****p < 0.0001, ns means not significant. The figures were Created with BioRender.com

The three ELISA assays evaluated (ID.VET, IDEXX, and Paralisa™) produced differing detection patterns across disease stages (Table 1, Supplementary Tables 2 and 3). The majority of subclinical animals was negative across all assays. In clinical animals, detection rates were markedly higher and Paralisa™ achieved the highest detection rate. The distribution of antibody responses detected by the three ELISA assays is shown in (Fig. 2a, b, c, and d). All animals from JD-free herds (*n* = 29) were ELISA negative (Supplementary Table 1).

### Diagnostic sensitivity, specificity, and agreement

Sensitivity and specificity estimated for each test using culture as the gold standard are presented in (Table 1). Paralisa™ showed the highest sensitivity followed by ID.VET. The IDEXX assay was significantly less sensitive (4%, 95% CI: 0.01–0.12%). Despite these differences, all three assays exhibited perfect specificity (100%, 95% CI: 0.89–1.0.89.0%). Agreement between assays varied as shown in Table 2. The IDEXX assay displayed poor agreement with both ID.VET and Paralisa™. In contrast, ID.VET and Paralisa™ demonstrated almost perfect agreement, confirming consistency in their diagnostic outputs. Combining the two serological assays ID.VET ELISA and the Paralisa™ provided no significant improvement for the diagnosis of Johne’s disease, as there was good agreement between the two assays (Parallel interpretation Sensitivity = 0.49 (0.37–0.61) and Specificity = 1 (0.89–1.89); Serial interpretation Sensitivity = 0.41 (0.3–0.53) and Specificity = 1 (0.89–1.89)).

### ROC curve analysis and determination of alternative cut-off values

High shedders generally had higher median optical density (OD) values or ELISA units well above the cutoffs while low shedders often had very low to borderline readings. The wide confidence intervals suggest a lot of variability in the latter group (Table 3).

ROC analysis demonstrated variable discriminatory performance among the assays (Supplementary Table 4). Paralisa™ (PPDj) achieved the highest overall of area under the curve (AUC) (83%, 95% CI:76–89%), followed closely by ID.VET (81%, 95% CI: 73–89%), indicating good diagnostic discrimination. The IDEXX and Paralisa™ (PPA) assays had lower AUCs than these two tests, showing limited performance (IDEXX AUC = 59%, 95% CI: 49–71%), (Paralisa™ PPA AUC = 61%, 95% CI: 51–71%).

Calculations of alternative cut-off values revealed OD values as low as 2 for the IDEXX assay and 0.41 for the PPDj Paralisa™ assay. While these alternative cut-offs may increase sensitivity, they are far too low to give a meaningful result in the actual test (Supplementary Table 4).

### Evaluation of ID.VET assay repeatability

Following exclusion of suspect results, the McNemar test p value was 0.66 suggesting that there was no significant difference in the agreement of test results between runs. The intraclass correlation coefficient (ICC) (3,1) was 0.932 (95% CI: 0.894–0.955, *p* < 0.001), demonstrating excellent reliability between test runs. Bland–Altman analysis revealed that the second set of OD values obtained from laboratory 2 (Supplementary Fig. 1) was on average higher than the first (mean difference 9.31, 95% CI: 3.71–14.91, *p =* 0.001), suggesting systematic bias (Supplementary Fig. 2). This bias increased with higher OD values, and the limits of agreement were wide, indicating greater variability at higher antibody titres.

## Discussion

The present study provides a comparative evaluation of three ELISA assays, two commercial assays (ID.VET and IDEXX) and one laboratory based assay (Paralisa™) for the detection of MAP-specific antibodies in serum collected from farmed sika deer for the first time. These findings provide valuable insights into the usefulness of serological assays for JD surveillance in wild ruminants, especially sika deer, which is crucial for identifying natural reservoirs of MAP^28–30^. As currently, no cervid-specific commercial ELISA or species-adapted conjugate is available for sika deer, these widely used commercially available multi-species ruminant ELISA kits were chosen for evaluation.

In this study, bacterial culture was used as the gold standard diagnostic method. Clinical signs in conjunction with the culture results were then used to classify the infection status of animal as JD-free, subclinical, or clinical. As expected, culture positivity was strongly associated with disease severity of clinically affected deer (100%) and subclinically infected deer (92.5%), while all JD-free deer were negative (0.0%). This confirms the progressive increase in bacterial burden with advancing disease, consistent with the pathogenesis of Johne’s disease^2,4,6,36^.

The automated liquid culture system for MAP isolation was chosen in this study as it has been reported to provide improved sensitivity and reduced time to detection compared with conventional solid media for MAP isolation in cattle and red deer^37,38^. Further, the para-JEM broth incubated in the VersaTREK system was successfully used when investigating subclinical MAP infection in captive white-tailed deer^2^.

When the ELISA assays were compared with culture, significant differences in diagnostic performance emerged. Analysis of antibody titres revealed a positive relationship between shedding intensity and serological response. Deer with high or very rapid culture growth, predominantly clinical animals, exhibited significantly elevated OD values in both Paralisa™ and ID.VET assays, clearly surpassing cut-off thresholds. By contrast, subclinical animals with low or very low culture growth often displayed weak or absent serological responses, overlapping with JD-free controls. This has previously been reported in red deer when using the Paralisa™ assay, where the positive predictive value increased with shedding thresholds^9^. Similar findings have been reported in cattle^23^, sheep^25^, and goat^39^. However, the fact that the IDEXX assay failed to detect even some high-shedding animals in subclinical and clinical affected sika deer assessed by Paralisa™ assay raises concerns about its utility in deer and reinforces the need for species-appropriate diagnostic tools.

Both Paralisa™ and ID.VET assays showed superior sensitivity compared with the IDEXX assay, especially in clinically affected deer. These differences likely arose from variations in antigen preparation, assay design, and conjugate specificity. The IDEXX assay, for example, was developed and validated primarily for cattle, sheep and goat using anti-bovine IgG conjugates, which may exhibit reduced binding affinity for deer immunoglobulins^10^. A previous study on white-tailed deer suggested that the poor performance of the IDEXX assay in white-tailed deer may be due to the lack of assay optimisation for this species, even though protein IgG conjugates have been shown to bind white-tailed deer immunoglobulins similarly to bovine immunoglobulin^8^. Therefore, these limitations in performance of the IDEXX assay in sika deer were not surprising, but the assay had been chosen for evaluation here because it is extensively validated in domestic ruminants, commercially available, broadly applied in MAP surveillance programs, and demonstrates well-characterised performance in related species. By contrast, Paralisa™ assay incorporates reagents tailored to deer immunoglobulins, which may explain its superior performance in this host species. Despite these differences in sensitivity, all assays maintained perfect or near-perfect specificity, with no false positives recorded apart from a single “suspect” classification by ID.VET assay. While false positive ELISA results have been reported in cattle infected with *Mycobacterium bovis*, exposed to environmental mycobacteria or tested within 14 days of the last tuberculin skin test^40,41^, none of these factors applied to the sika deer in our study.

Subclinically infected deer, however, were more difficult to detect serologically. This disparity likely reflects the immunological shift that occurs during disease progression. Clinically affected deer tend to exhibit robust humoral (Th2-type) responses and elevated antibody titres, making ELISA detection more reliable, whereas subclinical deer often remain in a predominantly cell-mediated (Th1-type) immune phase, resulting in weaker or undetectable antibody, while they may still shed MAP and contribute to transmission^27,42^. These results are consistent with reports in red deer, cattle and sheep, where ELISA sensitivity in subclinical animals typically ranges between 15% and 33%^9,23,43^. Such low sensitivity in early infection underscores the limitations of relying on serology alone for herd-level surveillance, particularly in species like deer where validation studies remain scarce. Nonetheless, the identification of even a subset of subclinical animals through serology remains valuable, as removal of these individuals can reduce environmental contamination and disease spread^2^.

The diagnostic performance observed in this study is broadly consistent with findings from other deer species. In farmed red deer, ELISA sensitivity against culture has been reported between 75 and 91%, with specificity typically exceeding 95%, supporting their usefulness in herd-level surveillance despite limited ability to detect all infected individuals^27^. Studies in fallow deer similarly demonstrated moderate sensitivity (62–88%) but very high specificity^26^. In white-tailed deer, commercial ELISAs such as the IDEXX assay showed poor sensitivity, comparable to the low values observed in our study, indicating that assays optimised for cattle may underperform in deer^8^. In elk, diagnostic agreement between serological assays has also been variable, highlighting the importance of assay adaptation to species-specific immune responses^10^.

The almost perfect agreement we observed between Paralisa™ and ID.VET assays suggests that both assays are detecting a comparable antibody response in sika deer, reinforcing their reliability for diagnostic use in this species. Combining the two serological assays ID.VET ELISA and the Paralisa™ provided no significant improvement for the diagnosis in clinical or subclinical cases. By contrast, the poor concordance of the IDEXX assay with either test reflects its markedly lower sensitivity, a trend consistent with earlier reports in different deer species^3,8,10^.

ROC analysis provided a more nuanced comparison of diagnostic performance, independent of fixed manufacturer cut-offs. The high diagnostic accuracy of Paralisa™ and ID.VET assay showed that these assays can reliably discriminate between infected and uninfected sika deer, whereas the IDEXX assay performed little better than chance.

Calculating the alternative cut-off for the ELISA tests used in this study in order to improve sensitivity only yielded values for the IDEXX and Paralisa™ assays that were too low to be practical. This was likely due to the limited sample size, particularly the small number of confirmed negative animals. In addition, these findings highlight the difficulty of identifying MAP infection in subclinical infected sika deer that have not mounted a detectable humoral immune response, while already shedding the bacteria.

The ID.VET ELISA demonstrated excellent repeatability, with high agreement across runs and no significant difference in classification between test runs in the two laboratories. However, a modest systematic bias emerged as the OD values in the second laboratory readings were on average higher, especially at elevated OD values, where variability increased highlighting the importance of procedural standardisation between laboratories. While there was no significant difference of the OD values between laboratories in the negative control samples, a bigger number of control samples would be needed for reliable concordance estimation. Possible contributing factors to the inter-laboratory OD variation include differences in test batches laboratory conditions, plate handling, or operator technique^44,45^.

This research study has several limitations that should be considered when interpreting the findings. First, the relatively small number of negative samples may have influenced estimates of diagnostic performance. In addition, sampling was restricted to a single JD-infected herd and one JD-free herd, limiting the generalisability of the findings. Broader validation across multiple herds, geographic regions, and management systems would strengthen confidence in the applicability of these results, as immune responses may vary under different environmental and epidemiological conditions. Another limitation is the cross-sectional design and single time-point sampling, which prevents assessment of antibody dynamics over the course of infection. Longitudinal studies following deer from early infection through clinical disease would provide valuable insight into antibody kinetics and temporal assay performance as already confirmed in the cattle^46–48^. Furthermore, the assays used in this study were originally designed for other species, and diagnostic accuracy in sika deer may be improved using species specific antibodies. Potential cross-reactivity with environmental mycobacteria cannot be excluded, but was unlikely given the absence of false negative results.

## Conclusion

This study provides the first comparative evaluation of three ELISA assays for the detection of MAP antibodies in farmed sika deer. While all assays demonstrated excellent specificity, the Paralisa™ and ID.VET assays offered superior sensitivity compared with the IDEXX assay, particularly in clinically affected and high-shedding sika deer. These findings support the use of Paralisa™ and ID.VET ELISAs as practical tools for JD monitoring, surveillance and control in infected sika deer herds, while highlighting the continuing challenges of reliably detecting subclinical infections.

## Materials and methods

### Informed consent

Informed consent was obtained from the owners of both herds prior to the commencement of the study.

### Animals and samples

**Fig. 3:**
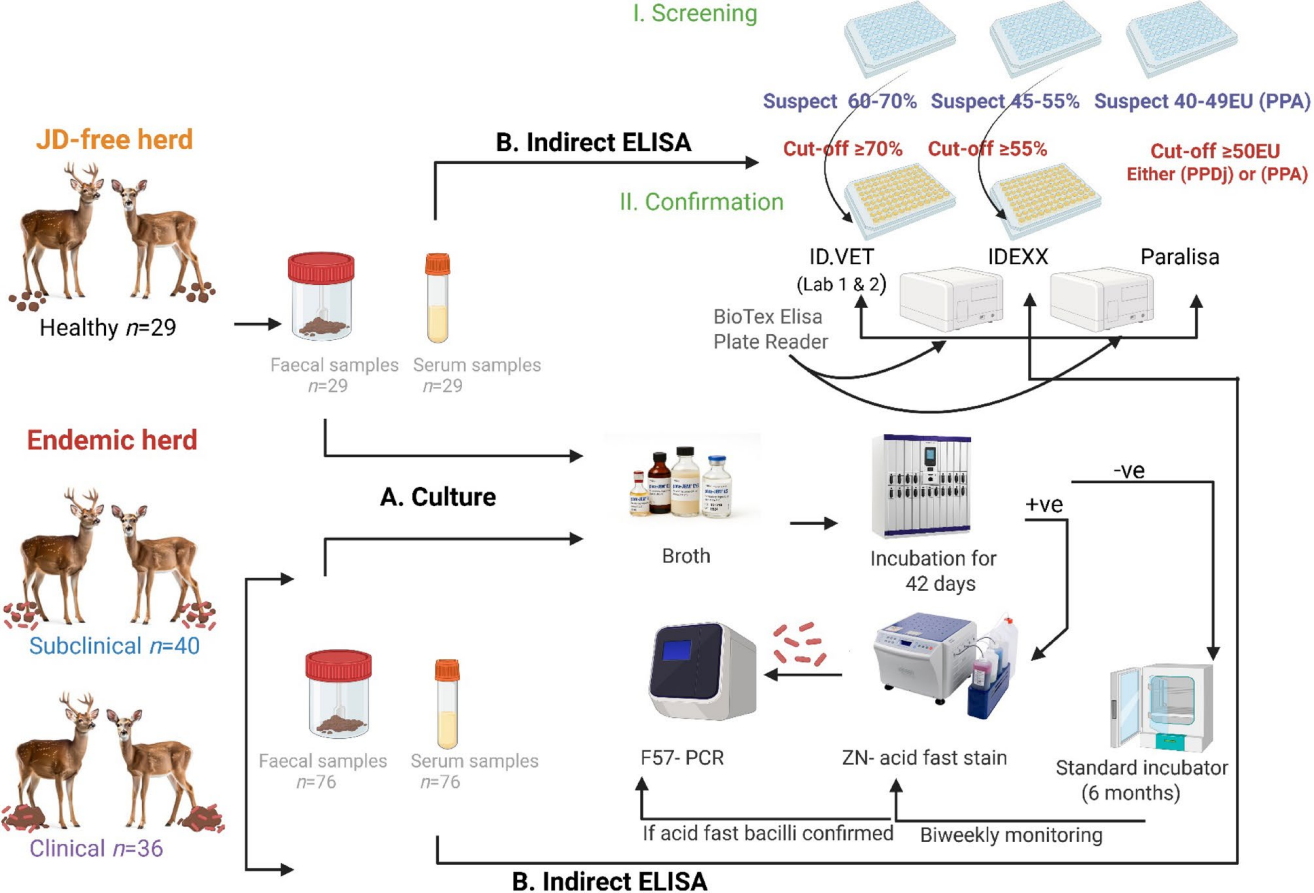
Overview of the animals and methodology used in this study. This figure was created with BioRender.com

A total of 105 paired faecal and serum samples from two herds were collected for this study. These comprised 76 samples from sika deer farmed commercially on a privately owned farm in Co. Waterford, Ireland. Thirty-six (47.36%) of these samples were obtained from clinically affected deer postmortem. The animals had been either found dead within 24 h or were euthanised by an intravenous overdose of Pentobarbital sodium (Euthasol^®^vet. 400 mg/ml). The remaining 40 samples (52.63%) were collected at slaughter from apparently healthy deer originating from the same infected herd. In addition, 29 samples were obtained postmortem from sika deer that died within 24 h due to other diseases or were culled by an intravenous overdose of Pentobarbital sodium (Euthasol^®^vet. 400 mg/ml) for management reasons from a JD-free herd located Co. Kildare, Ireland (Supplementary Fig. 3). Faecal samples (~ 20 g) were obtained per rectum using disposable gloves, transferred into sterile screw-cap containers, and immediately stored at −80 °C until processing. Blood samples (10 ml) were collected from the jugular vein antemortem, at slaughter or postmortem using sterile, anticoagulant-free vacutainer tubes. Following clotting, samples were centrifuged at 200 × g for 15 min to separate the serum. The recovered serum was aliquoted into 2 ml cryovials and stored at −80 °C until tested (Fig. 3).

### Faecal culture

All faecal samples were processed using the VersaTREK system (TREK para-JEM^®^; TREK Diagnostic Systems, Cleveland, OH, USA) as previously described^49^. A sample was declared positive after MAP was confirmed in signal positive samples by detecting acid-fast bacilli using Ziehl-Neelsen (ZN) staining and by a qPCR targeting the F57 locus of MAP^49^. The qPCR assay co-amplifies 16 S rDNA that is specific for the genus *Mycobacterium* and F57 locus for detection of MAP using Jump-Start Taq Ready Mix (Sigma-Aldrich) according to the manufacturer’s instructions. The shedding rate was determined by the time to signal positive appeared on VersaTREK system. Very high shedders < 6 days, high shedders cultured positive in < 20 days, moderate shedders in 21–28 days, low shedders in 29–42 days and very low shedders > 42 days^49,50^.

### Description of ELISA assays

All serum samples were tested using two commercially available ELISA assays (Supplementary Table 5). ID.VET (ID Screen^®^ Paratuberculosis Indirect Screening test, ID.VET, Grabels, France) used a purified MAP extract as a coating antigen and anti-ruminant IgG conjugate validated for detecting antibodies against MAP in cattle, sheep, and goats^26^. The IDEXX assay (IDEXX Paratuberculosis Screening Ab, Montpellier, France) also used a purified MAP extract as a coating antigen and anti-bovine IgG conjugate validated for detecting antibodies against MAP in cattle, sheep and goats^10^. Both ELISA assays were complemented by a verification assay to increase diagnostic reliability. In addition to these commercial assays, the Paralisa™ IgG1 ELISA specifically developed for red deer by the University of Otago at the Disease Research Limited (DRL) laboratory, New Zealand, was used (Supplementary Table 5). This assay uses two antigens, denatured purified protein derivative (PPDj) and protoplasmic antigen (PPA), combined with a deer-specific IgG1 conjugate to enhance detection sensitivity in red deer^27^. Results were expressed in ELISA units (EU), with a recommended cut-off of 50 EU. A sample was considered positive if either antigen produced a titre ≥ 50 EU, while results were classified as suspect when PPA values fell between 40 and 49 EU. Values below these thresholds were considered negative^9,27^.

### Commercial ELISA screening assays

The two commercial ELISA assays (ID.VET and IDEXX) were performed according to the manufacturer’s instructions in laboratory 1 (Ireland). Prior to testing, serum samples, positive and negative control sera supplied by the ELISA assays, were equilibrated to room temperature for 2 h. In addition, a positive control serum obtained from a sika deer with clinical JD was added. Serum samples and the additional positive control were analysed in single wells, while the commercial ELISA assays’ positive and negative control sera were analysed in duplicates (Supplementary Fig. 4). The OD values were measured at 450 nm (Agilent BioTek Microplate Readers), and results were interpreted based on the manufacturer’s cut-off values. Samples with sample-to-positive (S/P) ratios ≥ 70% (ID.VET) or ≥ 55% (IDEXX) were classified as positive, while those between 60 and < 70% (ID.VET) or 45% and < 55% (IDEXX) were considered ‘suspect’.

### Commercial ELISA verification assays

Samples classified as positive were subjected to verification using the ID.VET (ID Screen^®^ Paratuberculosis Indirect, Confirmation Test, ID.VET, Montpellier, France) and the IDEXX (IDEXX Paratuberculosis Verification Ab, Montpellier, France) assays, according to the manufacturer’s instructions. In this study, we also subjected all suspect samples to the same verification procedure to ensure accurate classification. Positive, negative, and additional positive control, along with test samples, were analysed in duplicate (Supplementary Fig. 3). The verification assays were conducted following the same procedures described for the initial ELISA screening. In addition, the same classification categories and cut-off values applied in the screening tests were also used for the verification tests. However, in these two assays, the result was read from the second column for each sample (Supplementary Fig. 4).

### Paralisa™ assay

All serum samples (*n* = 105) were shipped to DRL, Agresearch Invermay, Mosgiel 9092, New Zealand, (laboratory 2) for testing with the Paralisa™ IgG1 ELISA, an assay specifically developed for deer and previously described^9,27^. Following established protocols, results were expressed in EU, with a cut-off value of 50 EU applied as recommended.

### Definition of infection status

For the purposes of this study, infection status was defined using faecal culture as the gold standard method and confirmed by ZN staining and F57-PCR. Animals that were culture-positive for MAP were classified as infected, while culture-negative animals were considered uninfected. For serological testing, only the results obtained from ELISA verification assays (ID.VET and IDEXX) were considered definitive and were used to classify the immune status of each animal tested.

### Test repeatability

In order to evaluate test repeatability, the ID.VET assay was run on the 105 serum samples twice in two different laboratories (1 and 2). While the analysis was based on results from laboratory 1, the repeat run in laboratory 2 was used to examine systematic differences and variability between laboratories.

### Statistical analysis

All statistical analyses were conducted in R Studio 2025.09.0^51^ running R version 4.5.1^52^. The packages used for the analysis were blandr v.0.6.0, dplyr v.1.1.4, epiR v.2.0.87, irr v.0.84.1, ggplot v.4.0.0, pROC v1.19.0.1 and psych v.2.5.6^53–58^. This study applied the STRADAS-paraTB criteria that detail reporting standards for animal diagnostic accuracy studies for Johne’s disease^59^. Diagnostic test accuracy metrics obtained from the IDEXX and ID.VET tests run in laboratory 1 and the Paralisa™ (considering both antigens) in laboratory 2, including sensitivity, specificity, positive and negative predictive values, Youden’s Index and the diagnostic accuracy were calculated using faecal culture as the reference standard method. Animals classified as suspect by ELISA were excluded from the analyses.

Exact binomial 95% confidence intervals were estimated for sensitivity and specificity. Agreement between ELISA assays was also assessed using unweighted Cohen’s kappa statistics with 95% confidence intervals. Receiver operating characteristic (ROC) curves and the corresponding area under the curve (AUC) were generated to assess test discriminatory ability, with optimal cut-off values determined by maximising Youden’s Index. AUC for the Paralisa assay was calculated for each antigen PPDi and PPA separately to establish individual alternative cut-off points. Non-parametric tests, including the Mann–Whitney U test and Dunn’s post hoc test following Kruskal-Wallis analysis, were applied to compare OD values across groups and shedding categories. Correlations between continuous OD values were assessed using Kendall’s τ coefficient. The test repeatability of the ID.VET ELISA between the two laboratories was assessed using three separate metrics. McNemar test was used to assess the agreement in test classification between the laboratories. The ICC estimate and 95% confidence intervals were calculated based on a single measurement, absolute-agreement, 2-way mixed-effects model to evaluate the reliability of the test measurements. Bland Altmann plots were constructed to visualize the differences in OD values between test runs and assess for systemic bias.

**Table 1:** Serological detection of MAP antibodies by three ELISAs in subclinically and clinically MAP-infected sika deer.

ELISA assays	ID.VET			IDEXX			Paralisa™		
Diagnostic performance	Se 43% (31-55%)			Se 4% (1-12%)			Se 48 % (36-60%)		
	Sp 100% (89-100%)			Sp 100% (89-100%)			Sp 100% (89-100%)		

Disease status	POS	SUS	NEG	POS	SUS	NEG	POS	SUS	NEG
Subclinical (*n* = 40)	6 (15%)	1 (2.5%)	33 (82.5%)	1 (2.5%)	0 (0%)	39 (97.5%)	6 (15%)	0 (0%)	34 (85.0%)
Clinical (*n* = 36)	25 (69.44%)	0 (0%)	11 (30.55%)	2 (5.55%)	1 (2.77%)	33 (91.66%)	29 (80.55%)	0 (0%)	7 (19.44%)

Se = Sensitivity, Sp = Specificity

**Table 2:** Total agreements and Cohen’s kappa statistics and 95% confidence intervals (CI) of the three ELISAs.

Types of anti-MAP assays compared	Cohen’s kappa statistic*	Strength of agreement	Lower 95% confidence intervals	Upper 95% confidence intervals
IDEXX vs. ID.VET	0.14	poor	0.00	0.28
IDEXX vs. Paralisa™	0.11	poor	−0.01	0.24
ID.VET vs. Paralisa™	0.86	almost perfect	0.76	0.97

*<0.20, poor; 0.21–0.40, fair; 0.41–0.60, moderate; 0.61–0.80, substantial (good); and 0.81–100, almost perfect agreement.

**Table 3:** Median ELISA reads in relation to culture results and different shedding rates.

ELISA	Culture shedding rate			
	No *n* = 32	Low *n* = 30	Moderate *n* = 5	High *n* = 38
IDEXX Neg	0.1* (−1.62-3.63)	NA	NA	NA
IDEXX Pos	NA	−1.3 (−2.31-41.19)	0 (−0.27-32.74)	23.35 (−1.34-58.81)
ID.VET Neg	0.85 (−2.22-18.93) *	NA	NA	NA
ID.VET Pos	NA	2.7 (−0.03-219.09)	2.9 (0.26–160.63.26.63)	134.95 (0.36–233.48.36.48)
Paralisa™ PPDj Neg	0 (−0.03-19.42) **	NA	NA	NA
Paralisa™ PPDj Pos	NA	0 (0–67.55.55)	13.45 (0.2–52.2)	57.5 (0–115)
Paralisa™ PPA Neg	3 (0–22.67.67) **	NA	NA	NA
Paralisa™ PPA Pos	NA	0 (0–65)	8 (0–33.5.5)	26 (0–120.05.05)

* Median OD value, 95% confidence interval, **median ELISA unit, n = number of animals tested.

## Supplementary Information

Below is the link to the electronic supplementary material.

Supplementary Material 1

## Data Availability

All data generated or analysed during this study are included in this published article and its supplementary information files.
