# Correction: Evaluation of three ELISA assays for detection of *Mycobacterium avium* subsp. *Paratuberculosis* antibodies in serum of sika deer (*Cervus nippon*)

**DOI:** 10.1038/s41598-026-65671-6

**Published:** 2026-08-11

**Authors:** Nasser Alotaibi, Emily A. Courcier, Máire C. McElroy, Simon Liggett, Hanne Jahns

**Affiliations:** 1 Department of Pathology, College of Veterinary Medicine, King Faisal University, 31982 Al-Ahsa, Saudi Arabia; 2 Pathobiology Section, School of Veterinary Medicine, Veterinary Science Centre, University College Dublin, Belfield, Dublin 4, D04W6F6 Ireland; 3 Centre for Veterinary Epidemiology and Risk Analysis, University College Dublin, Dublin, Ireland; 4 Central Veterinary Research Laboratory, Department of Agriculture, Food and the Marine, Celbridge, Kildare Ireland; 5 Disease Research Ltd, Mosgiel, New Zealand

Correction to: *Scientific Reports* 10.1038/s41598-026-51629-1, published online 19 May 2026

The original version of this Article contained an error in Table 1, where in the second column, the sensitivity for IDEXX assay was incorrect,

**Table 1:** Serological detection of MAP antibodies by three ELISAs in subclinically and clinically MAP-infected sika deer.

ELISA assays	ID.VET	IDEXX	Paralisa™
Diagnostic performance	Se 43% (31–55%)	Se 43% (31–55%)	Se 48% (36–60%)
	Sp 100% (89–100%)	Sp 100% (89–100%)	Sp 100% (89–100%)

Disease status	POS	SUS	NEG	POS	SUS	NEG	POS	SUS	NEG
Subclinical (n = 40)	6 (15%)	1 (2.5%)	33 (82.5%)	1 (2.5%)	0 (0%)	39 (97.5%)	6 (15%)	0 (0%)	34 (85.0%)
Clinical (n = 36)	25 (69.44%)	0 (0%)	11 (30.55%)	2 (5.55%)	1 (2.77%)	33 (91.66%)	29 (80.55%)	0 (0%)	7 (19.44%)

Se = Sensitivity, Sp = Specificity.

“43%”

now reads:

“4%”.

The original Article has been corrected.

