# Supplementary material for: Evaluation of three ELISA assays for detection of Mycobacterium avium subsp. paratuberculosis antibodies in serum of sika deer (Cervus nippon)
Source: Sci Rep. 2026 May 19;16:22746. doi: 10.1038/s41598-026-51629-1 (PMC13385845; doi:10.1038/s41598-026-51629-1)
Supplement: Supplementary file 1 — Supplementary Material 1 [file 41598_2026_51629_MOESM1_ESM.docx]

**Supplementary Table 1.** Details on faecal MAP culture and ELISA assays results of healthy sika deer.

| **Case ID** | **Origin** | **Animals**  **status** | **Faecal MAP culture results** | **Time to positivity (days)** | **Shedding rate** | **ID.VET**  **results**  **(lab 1)** | **S/P**  **(70%)** | **ID.VET**  **results**  **(lab 2)** | **S/P**  **(70%)** | **IDEXX results** | **S/P (55%)** | **Paralisa** **results** | **PPDj (50EU)** | **PPA (50EU)** |
| --- | --- | --- | --- | --- | --- | --- | --- | --- | --- | --- | --- | --- | --- | --- |
| 401/18 | Kildare | Healthy | Neg | 0 | No | Neg | -2.2 | Neg | 14 | Neg | 1.2 | Neg | 0 | 3 |
| 402/18 | Kildare | Healthy | Neg | 0 | No | Neg | -1 | Neg | 5 | Neg | 0.1 | Neg | 0 | 0 |
| 407/18 | Kildare | Healthy | Neg | 0 | No | Neg | -2.3 | Neg | 6 | Neg | 0.1 | Neg | 0 | 3 |
| 408/18 | Kildare | Healthy | Neg | 0 | No | Neg | 0.1 | Neg | 10 | Neg | 0.2 | Neg | 0 | 0 |
| 409/18 | Kildare | Healthy | Neg | 0 | No | Neg | -0.6 | Neg | 8 | Neg | 0 | Neg | 0 | 2 |
| 413/18 | Kildare | Healthy | Neg | 0 | No | Neg | 1.4 | Neg | 7 | Neg | 0.2 | Neg | 0 | 13 |
| 414/18 | Kildare | Healthy | Neg | 0 | No | Neg | 0.7 | Neg | 0 | Neg | 0.3 | Neg | 0 | 9 |
| 415/18 | Kildare | Healthy | Neg | 0 | No | Neg | 0.3 | Neg | 0 | Neg | 0 | Neg | 0 | 4 |
| 492/19 | Kildare | Healthy | Neg | 0 | No | Neg | 2.6 | Neg | 0 | Neg | 0.3 | Neg | 0 | 6 |
| 493/19 | Kildare | Healthy | Neg | 0 | No | Neg | -0.5 | Neg | 0 | Neg | 0.9 | Neg | 0 | 25 |
| 495/19 | Kildare | Healthy | Neg | 0 | No | Neg | -0.2 | Neg | 0 | Neg | 0.2 | Neg | 0 | 2 |
| 456/19 | Kildare | Healthy | Neg | 0 | No | Neg | 0.7 | Neg | 0 | Neg | 0 | Neg | 0 | 4 |
| 457/19 | Kildare | Healthy | Neg | 0 | No | Neg | 3.6 | Neg | 0 | Neg | 0.1 | Neg | 0 | 5 |
| 458/19 | Kildare | Healthy | Neg | 0 | No | Neg | 2.4 | Neg | 0 | Neg | 0 | Neg | 0 | 4 |
| 355/21 | Kildare | Healthy | Neg | 0 | No | Neg | 3.3 | Neg | 0 | Neg | 1.5 | Neg | 0 | 6 |
| 301/22 | Kildare | Healthy | Neg | 0 | No | Neg | 8.4 | Neg | 0 | Neg | 0.7 | Neg | 0 | 0 |
| 302/22 | Kildare | Healthy | Neg | 0 | No | Neg | 0.9 | Neg | 0 | Neg | 0 | Neg | 0 | 2 |
| 331/23 | Kildare | Healthy | Neg | 0 | No | Neg | 3.2 | Neg | 0 | Neg | 0.5 | Neg | 16.99 | 7 |
| 13/22 | Kildare | Healthy | Neg | 0 | No | Neg | 8.3 | Neg | 0 | Neg | 0 | Neg | 27.8 | 1 |
| 15/22 | Kildare | Healthy | Neg | 0 | No | Neg | 5.6 | Neg | 0 | Neg | 0 | Neg | 0 | 3 |
| 20/22 | Kildare | Healthy | Neg | 0 | No | Neg | 0.8 | Neg | 3 | Neg | 0 | Neg | -0.13 | 0 |
| 23/22 | Kildare | Healthy | Neg | 0 | No | Neg | 0 | Neg | 12 | Neg | 10.3 | Neg | 0 | 3 |
| 35/22 | Kildare | Healthy | Neg | 0 | No | Neg | 2.3 | Neg | 5 | Neg | 0.5 | Neg | 0 | 0 |
| 36/22 | Kildare | Healthy | Neg | 0 | No | Neg | -1.2 | Neg | 0 | Neg | -0.1 | Neg | 0 | 0 |
| 38/22 | Kildare | Healthy | Neg | 0 | No | Neg | 0.2 | Neg | 0 | Neg | 0.1 | Neg | 0 | 0 |
| 39/22 | Kildare | Healthy | Neg | 0 | No | Neg | 4.7 | Neg | 0 | Neg | 0.3 | Neg | 0 | 0 |
| 39*/22 | Kildare | Healthy | Neg | 0 | No | Neg | 5.7 | Neg | 0 | Neg | 0.7 | Neg | 0 | 12 |
| 40/22 | Kildare | Healthy | Neg | 0 | No | Neg | 1.3 | Neg | 0 | Neg | 0 | Neg | 0 | 22 |
| 41/22 | Kildare | Healthy | Neg | 0 | No | Neg | -1 | Neg | 0 | Neg | 1.7 | Neg | 0 | 18 |

**Supplementary Table 2.** Details on faecal MAP culture and ELISA assays results of subclinically affected sika deer.

| **Case ID** | **Origin** | **Animals**  **status** | **Faecal MAP culture results** | **Time to positivity (days)** | **Shedding rate** | **ID.VET**  **results**  **(lab 1)** | **S/P**  **(70%)** | **ID.VET**  **results**  **(lab 2)** | | **S/P**  **(70%)** | | **IDEXX**  **results** | **S/P (55%)** | **Paralisa**  **results** | **PPDj**  **(50EU)** | **PPA**  **(50EU)** |
| --- | --- | --- | --- | --- | --- | --- | --- | --- | --- | --- | --- | --- | --- | --- | --- | --- |
| S 1/16 | Waterford | Subclinical | POS | 36 | low | Neg | 1.4 | Neg | | 0 | | Neg | -1.3 | Neg | 0 | 0 |
| S 2/16 | Waterford | Subclinical | POS | 67 | very low | Neg | 3.6 | Neg | | 0 | | Neg | -1.2 | Neg | 0 | 0 |
| S 3/16 | Waterford | Subclinical | POS | 29.2 | low | Neg | 3.2 | Neg | | 0 | | Neg | -1.3 | Neg | 0 | 0 |
| S 4/16 | Waterford | Subclinical | POS | 42 | low | POS | 193.1 | POS | | 148 | | Neg | 22.1 | POS | 56 | 21 |
| S 5/16 | Waterford | Subclinical | POS | 20 | high | Neg | 3.2 | Neg | | 0 | | Neg | -0.5 | Neg | 0 | 0 |
| S 6/16 | Waterford | Subclinical | POS | 30 | low | POS | 224.6 | POS | | 228 | | Neg | 34.4 | POS | 12 | 65 |
| S 7/16 | Waterford | Subclinical | POS | 42 | low | Neg | 0.3 | Neg | | 0 | | Neg | 0.8 | Neg | 1 | 0 |
| S 8/16 | Waterford | Subclinical | POS | 15 | high | Neg | 1.5 | Neg | | 0 | | Neg | -0.9 | Neg | 1 | 0 |
| S 9/16 | Waterford | Subclinical | POS | 42 | low | Neg | 3.6 | Neg | | 0 | | Neg | -0.9 | Neg | 1 | 0 |
| S 10/16 | Waterford | Subclinical | POS | 42 | low | Neg | 4.6 | Neg | | 0 | | Neg | -1.8 | Neg | 2 | 0 |
| S 11/16 | Waterford | Subclinical | POS | 67 | very low | Neg | 2.3 | Neg | | 0 | | Neg | -2 | Neg | 13.27 | 20.39 |
| S 12/16 | Waterford | Subclinical | Neg | 0 | Non | Neg | 1.9 | Neg | | 6 | | Neg | -1.7 | Neg | 4 | 0 |
| S 13/16 | Waterford | Subclinical | POS | 33 | low | Neg | 0.6 | Neg | | 0 | | Neg | -0.9 | Neg | 0 | 0 |
| S 14/16 | Waterford | Subclinical | Neg | 0 | Non | Neg | 0.6 | Neg | | 0 | | Neg | -1.5 | Neg | 0 | 0 |
| S 15/16 | Waterford | Subclinical | POS | 12 | high | POS | 202.7 | POS | | 105 | | Neg | 18.7 | POS | 25 | 54 |
| S 16/16 | Waterford | Subclinical | POS | 15 | high | Neg | 34.2 | Neg | | 17 | | Neg | 0 | Neg | 1 | 0 |
| S 17/16 | Waterford | Subclinical | POS | 26.9 | moderate | SUS | 61 | Neg | | 34 | | Neg | 16.9 | Neg | 2 | 0 |
| S 18/16 | Waterford | Subclinical | POS | 42 | low | Neg | -0.1 | Neg | | 0 | | Neg | 2.3 | POS | 1 | 50 |
| S 19/16 | Waterford | Subclinical | POS | 12 | high | Neg | 0.4 | Neg | | 0 | | Neg | -1.3 | Neg | 8 | 0 |
| S 20/16 | Waterford | Subclinical | POS | 18 | high | POS | 203.2 | POS | | 216 | | Neg | 29.5 | POS | 18 | 65 |
| S 21/16 | Waterford | Subclinical | POS | 16 | high | Neg | 11.3 | Neg | | 2 | | Neg | -0.6 | Neg | 0.82 | 7.84 |
| S 22/16 | Waterford | Subclinical | Neg | 0 | Non | Neg | 1.7 | Neg | | 0 | | Neg | -1.6 | Neg | 0 | 0 |
| S 23/16 | Waterford | Subclinical | POS | 28 | moderate | Neg | 2.2 | Neg | | 0 | | Neg | 0 | Neg | 0 | 0 |
| S 24/16 | Waterford | Subclinical | POS | 33 | low | Neg | 0.8 | Neg | | 0 | | Neg | -2.2 | Neg | 0 | 0 |
| S 25/16 | Waterford | Subclinical | POS | 42 | low | Neg | 28.1 | | Neg | | 0 | Neg | -1.7 | Neg | 0 | 0 |
| S 26/16 | Waterford | Subclinical | POS | 67 | very low | Neg | 0.2 | | Neg | | 0 | Neg | -1.9 | Neg | 0 | 0 |
| S 27/16 | Waterford | Subclinical | POS | 42 | low | Neg | 24 | | Neg | | 0 | Neg | -1.9 | Neg | 0 | 0 |
| S 28/16 | Waterford | Subclinical | POS | 33 | low | Neg | 2.5 | | Neg | | 5 | Neg | -2.6 | Neg | 0 | 0 |
| S 29/16 | Waterford | Subclinical | POS | 81 | very low | Neg | 1.1 | | Neg | | 0 | Neg | -1.5 | Neg | 0 | 0 |
| S 30/16 | Waterford | Subclinical | POS | 42 | low | Neg | 0.9 | | Neg | | 0 | Neg | -1.9 | Neg | 0 | 0 |
| S 31/16 | Waterford | Subclinical | POS | 17 | high | POS | 134.9 | | SUS | | 66 | Neg | 1.6 | Neg | 23 | 31 |
| S 32/16 | Waterford | Subclinical | POS | 12 | high | Neg | -0.2 | | Neg | | 0 | Neg | -1.8 | Neg | 0 | 0 |
| S 33/16 | Waterford | Subclinical | POS | 30 | low | POS | 217 | | POS | | 297 | POS | 59.1 | POS | 98 | 65 |
| S 34/16 | Waterford | Subclinical | POS | 32 | low | Neg | 9.4 | | Neg | | 0 | Neg | -1.8 | Neg | 2.17 | 2.76 |
| S 35/16 | Waterford | Subclinical | POS | 34 | low | Neg | 8.19 | | Neg | | 0 | Neg | -1.8 | Neg | 0 | 0 |
| S 36/16 | Waterford | Subclinical | POS | 33 | low | Neg | 3.1 | | Neg | | 10 | Neg | -1.3 | Neg | 0 | 0 |
| S 37/16 | Waterford | Subclinical | POS | 34 | low | Neg | 2.2 | | Neg | | 2 | Neg | -1.3 | Neg | 0 | 0 |
| S 38/16 | Waterford | Subclinical | POS | 42 | low | Neg | 0.2 | | Neg | | 0 | Neg | -1.2 | Neg | 0 | 0 |
| S 39/16 | Waterford | Subclinical | POS | 42 | low | Neg | 0 | | Neg | | 0 | Neg | -1.5 | Neg | 0 | 0 |
| S 40/16 | Waterford | Subclinical | POS | 81 | very low | Neg | 1.6 | | Neg | | 0 | Neg | -1.3 | Neg | 0 | 0 |

**Supplementary Table 3.** Details on faecal MAP culture and ELISA assays results of clinically affected sika deer.

| **Case**  **ID** | **Origin** | **Animals**  **status** | **Faecal MAP culture results** | **Time to positivity**  **(days)** | **Shedding**  **rate** | **ID.VET results**  **(lab1)** | **S/P**  **(70%)** | **ID.VET results**  **(lab2)** | **S/P**  **(70%)** | **IDEXX**  **results** | **S/P**  **(55%)** | **Paralisa**  **results** | **PPDj**  **(50EU)** | **PPA**  **(50EU)** |
| --- | --- | --- | --- | --- | --- | --- | --- | --- | --- | --- | --- | --- | --- | --- |
| 533/16 | Waterford | Clinical | POS | 14 | high | Neg | 54 | Neg | 29 | Neg | 18.1 | POS | 70.45 | 10 |
| 534/16 | Waterford | Clinical | POS | 11 | high | POS | 125.2 | POS | 102 | Neg | 14.4 | POS | 20.18 | 53 |
| 385/17 | Waterford | Clinical | POS | 44 | low | Neg | 5.9 | Neg | 0 | Neg | 0.6 | Neg | 32.82 | 16 |
| 386/17 | Waterford | Clinical | POS | 27 | moderate | Neg | 4.8 | Neg | 15 | Neg | -0.3 | Neg | 13.45 | 11 |
| 387/17 | Waterford | Clinical | POS | 11 | high | POS | 220.4 | POS | 242 | Neg | 38.5 | POS | 115 | 55 |
| 388/17 | Waterford | Clinical | POS | 5 | very high | POS | 233.1 | POS | 253 | Neg | 43.5 | POS | 115 | 71 |
| 389/17 | Waterford | Clinical | POS | 7 | high | POS | 135.4 | POS | 89 | Neg | 11.1 | POS | 62 | 0 |
| 390/17 | Waterford | Clinical | POS | 11 | high | POS | 81.9 | Neg | 49 | Neg | 6.2 | POS | 66 | 3 |
| 391/17 | Waterford | Clinical | POS | 10 | high | POS | 231.5 | POS | 256 | SUS | 49.9 | POS | 102 | 51 |
| 573/17 | Waterford | Clinical | POS | 42 | low | Neg | 2.9 | Neg | 0 | Neg | 0.4 | Neg | 2 | 0 |
| 574/17 | Waterford | Clinical | POS | 32 | low | Neg | 8.1 | Neg | 0 | Neg | 0 | Neg | 4 | 2 |
| 575/17 | Waterford | Clinical | POS | 10 | high | POS | 126.9 | POS | 101 | Neg | 21.9 | POS | 52.39 | 18.56 |
| 576/17 | Waterford | Clinical | POS | 11 | high | POS | 150 | POS | 108 | Neg | 26.4 | POS | 105 | 14 |
| 577/17 | Waterford | Clinical | POS | 12 | high | POS | 229.5 | POS | 189 | Neg | 42.5 | POS | 43 | 65 |
| 578/17 | Waterford | Clinical | POS | 12 | high | POS | 230.2 | POS | 160 | Neg | 39.4 | POS | 80 | 65 |
| 579/17 | Waterford | Clinical | POS | 10 | high | POS | 233.4 | POS | 280 | POS | 63.9 | POS | 111 | 133 |
| 580/17 | Waterford | Clinical | POS | 10 | high | POS | 123.94 | POS | 92 | Neg | 23.9 | POS | 75 | 5 |
| 581/17 | Waterford | Clinical | POS | 28 | moderate | Neg | 2.9 | Neg | 1 | Neg | 0 | Neg | 18 | 8 |
| 459/17 | Waterford | Clinical | POS | 10 | high | POS | 202.2 | POS | 150 | Neg | 29.1 | POS | 89 | 76 |
| 462/17 | Waterford | Clinical | POS | 10 | high | POS | 177.4 | POS | 138 | Neg | 22.8 | POS | 79 | 21 |
| 463/17 | Waterford | Clinical | POS | 42 | low | Neg | 4.1 | Neg | 22 | Neg | 2.7 | POS | 0 | 53 |
| 464/17 | Waterford | Clinical | POS | 13 | high | POS | 184.1 | POS | 117 | Neg | 34.1 | POS | 79 | 30 |
| 465/17 | Waterford | Clinical | POS | 11 | high | POS | 228.1 | POS | 136 | Neg | 41.2 | POS | 101 | 119 |
| 251/17 | Waterford | Clinical | POS | 10 | high | POS | 234.5 | POS | 215 | POS | 58.4 | POS | 113 | 34 |
| 252/17 | Waterford | Clinical | POS | 17 | high | POS | 232.1 | POS | 279 | Neg | 34.6 | POS | 102 | 115 |
| 253/17 | Waterford | Clinical | POS | 12 | high | POS | 162.7 | POS | 138 | Neg | 21.9 | POS | 31 | 54 |
| 254/17 | Waterford | Clinical | POS | 21 | moderate | POS | 171.7 | POS | 141 | Neg | 34.5 | POS | 56 | 36 |
| 255/17 | Waterford | Clinical | POS | 13 | high | POS | 110 | Neg | 58 | Neg | 29.8 | POS | 70 | 3 |
| 256/17 | Waterford | Clinical | POS | 15 | high | POS | 169.7 | POS | 101 | Neg | 20.6 | POS | 29 | 61 |
| 95/17 | Waterford | Clinical | POS | 9 | high | Neg | 49.9 | Neg | 52 | Neg | 3.3 | POS | 51.63 | 15.15 |
| 96/17 | Waterford | Clinical | POS | 12 | high | POS | 126.4 | SUS | 62 | Neg | 34.6 | POS | 36 | 65 |
| 02712/17 | Waterford | Clinical | POS | 9 | high | POS | 205.8 | POS | 143 | Neg | 31.6 | POS | 98 | 14 |
| 02713/17 | Waterford | Clinical | POS | 8 | high | POS | 91.7 | Neg | 48 | Neg | 31.4 | POS | 53 | 22 |
| 02714/17 | Waterford | Clinical | POS | 11 | high | Neg | 49 | Neg | 4 | Neg | 11 | Neg | 10 | 4 |
| 02715/17 | Waterford | Clinical | POS | 11 | high | Neg | 56.3 | Neg | 37 | Neg | 27.4 | Neg | 36 | 5 |
| 182/18 | Waterford | Clinical | POS | 10 | high | Neg | 22.9 | Neg | 43 | Neg | 1.6 | POS | 109.38 | 42.44 |

**Supplementary Table 4.** Receiver operating characteristic (ROC) curves and 95 % confidence intervals (CI), optimal cut-off value, sensitivity and specificity values of the three ELISA assays.

| **Diagnostic parameters** | **ID.VET** | **IDEXX** | **Paralisa**^TM^ | |
| --- | --- | --- | --- | --- |
|  |  |  | **PPDj** | **PPA** |
| **ROC (95%CI)** | 81% (73-89%) | 59% (49-71%) | 83% (76-89%) | 61% (51%-71%) |
| **Optimal cut-off value** | 19.25 | 2 | 0.41 | 13.5 |
| **Sensitivity (95%CI)** | 59% (44-85%) | 52% (38-64%) | 71% (59-80%) | 45% (27-59%) |
| **Specificity (95%CI)** | 97% (68-100%) | 100% (91-100%) | 91% (81-100%) | 94% (81-100%) |

**
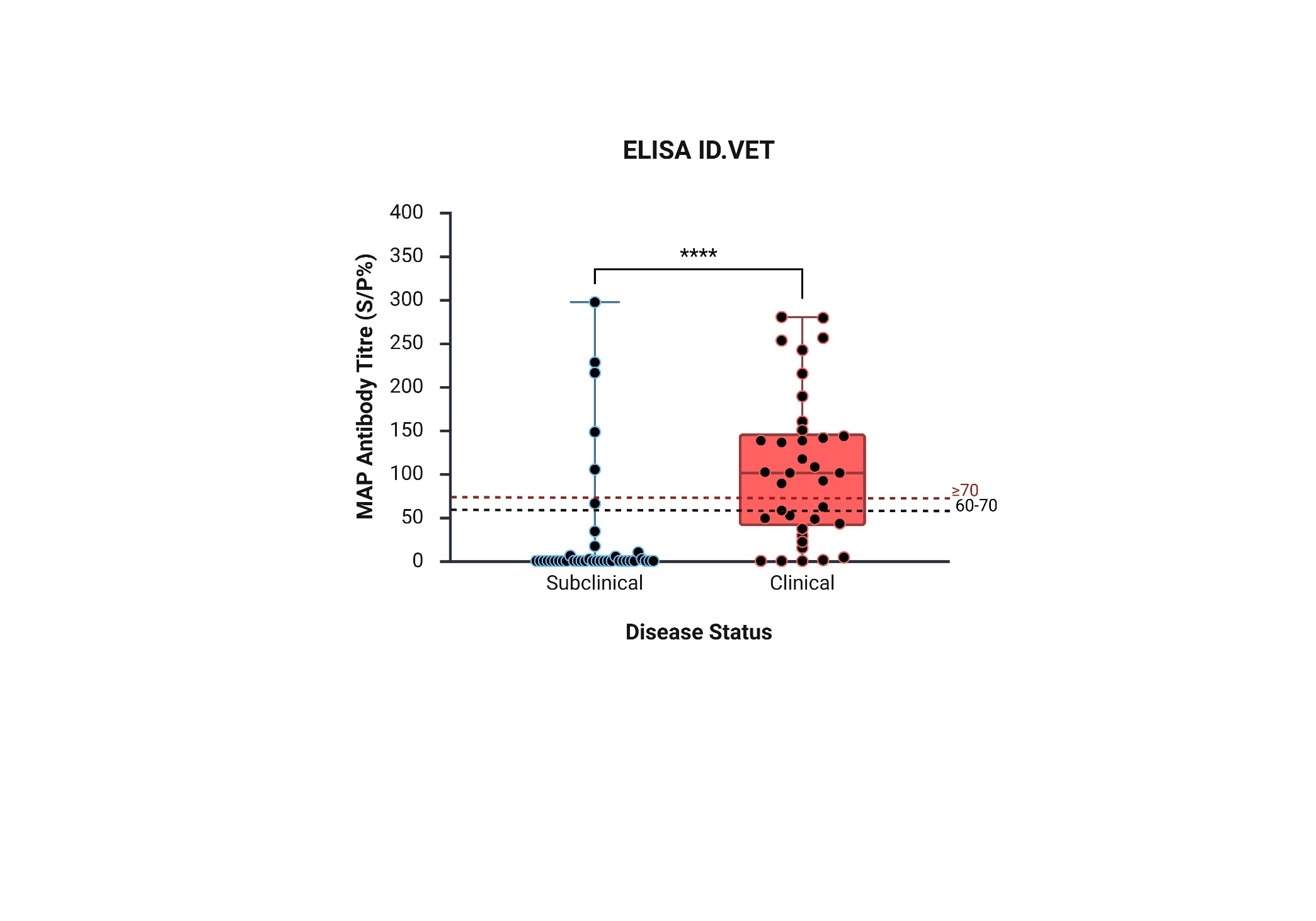
**

**Supplementary Figure 1**. Boxplot showing the detection of antibodies titres to MAP by ID.VET ELISA assay (lab 2). The dashed line (red) indicates the positive cut-off value of test. The dashed line (black) indicates the suspect (doubtful) cut-off value of test. Each spot represents one animal tested. Statistical significance was determined via the two-tailed Mann-Whitney U Test, *****p <*0.0001. Created with BioRnder.com


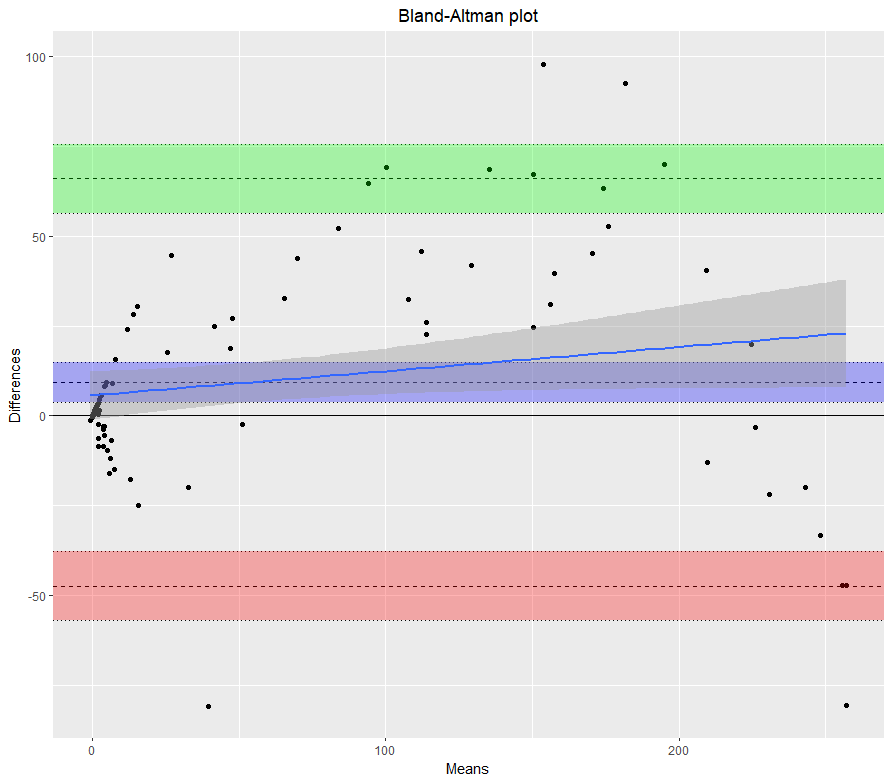


**Supplementary Figure 2.** Bland–Altman plot comparing the evaluation of ID.VET assay repeatability (Lab 1 and 2).


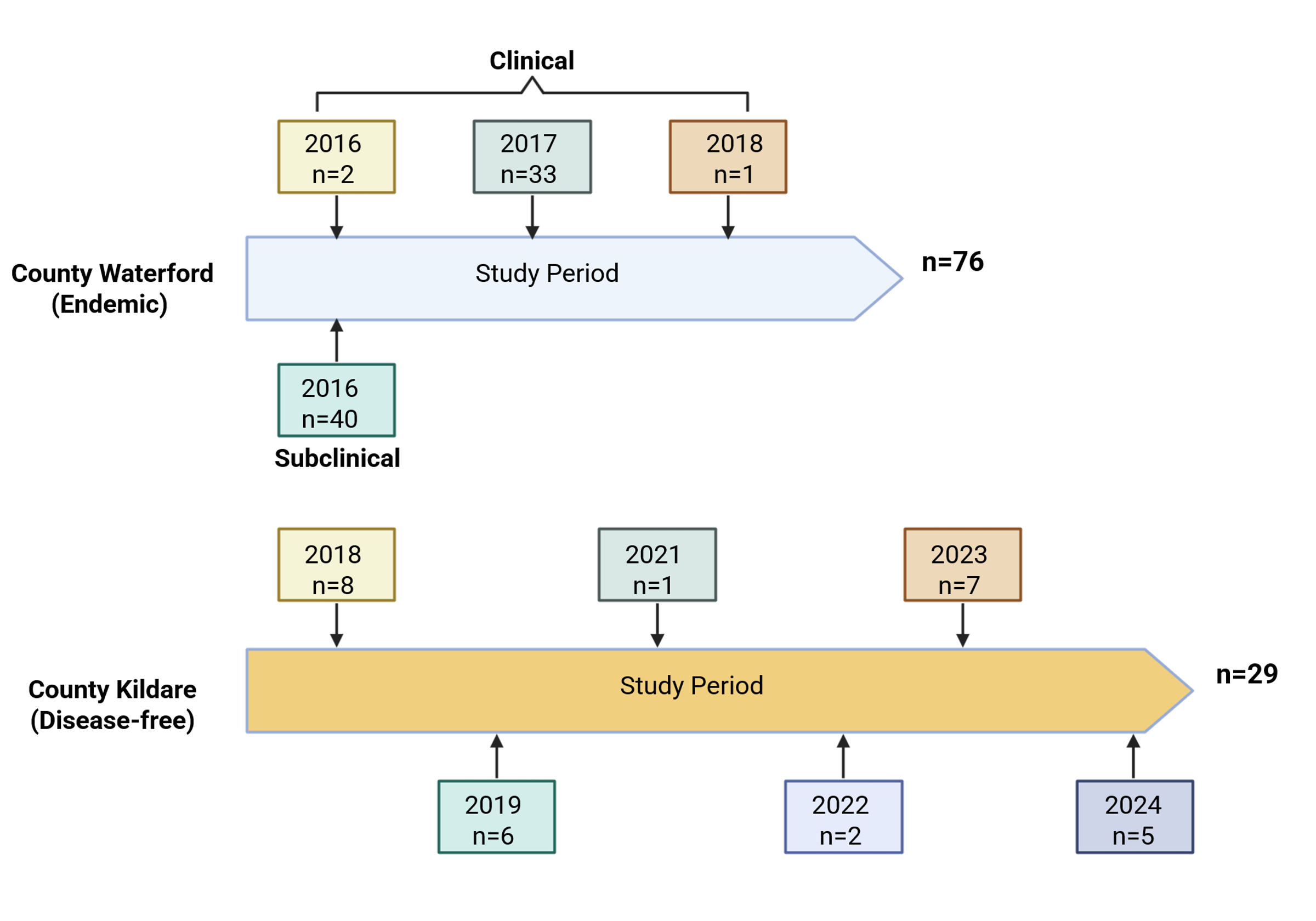


**Supplementary Figure 3.** Timeline of serum sample collection from endemic and JD-free herds during this study. Created in BioRender.com


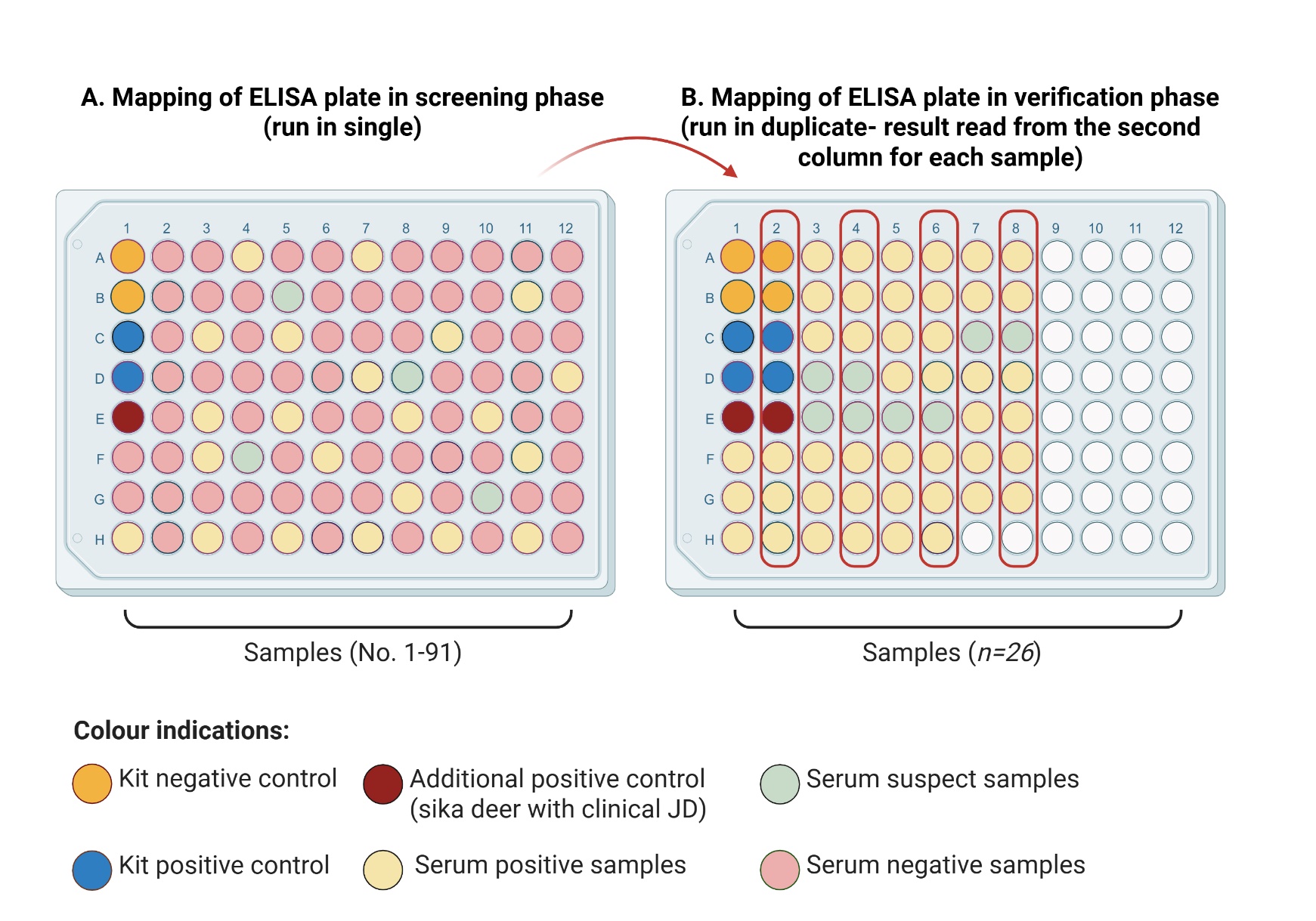


**Supplementary Figure 4.** ELISA 96-well plate layouts for screening and verification phases of serum sample testing. The figure shows the distribution of controls and serum samples in the two ELISA assays. Created in BioRender.com

**Supplementary Table 5.** Characteristics of the three ELISAs used in this study.

| **Test** | **Manufacturer** | **Testing format** | **Species for which the test was designed** | **Conjugate** | **Results interpretation criteria** |
| --- | --- | --- | --- | --- | --- |
| **ID.VET** | ID vet, France | Indirect ELISA | Cattle, sheep, goat | Anti-ruminant | Negative: ≤60%  Suspect: 60-70%  Positive: >70% |
| **IDEXX** | IDEXX, France | Indirect ELISA | Cattle, sheep, goat | Anti-bovine | Negative: ≤45%  Suspect: 45-55%  Positive: >55% |
| **Paralisa**^TM^ | New Zealand  (In-house) | Indirect ELISA | Red deer | Anti-deer | Negative: ≤40EU  Suspect: if the PPA is between 40-49EU  Positive: >50EU (If either of two proteins (PPDj & PPA) has an antibody titre over 50EU |
